# PD-L1 immunohistochemistry in non-small-cell lung cancer: unraveling differences in staining concordance and interpretation

**DOI:** 10.1007/s00428-020-02976-5

**Published:** 2020-12-04

**Authors:** Cleo Keppens, Elisabeth MC Dequeker, Patrick Pauwels, Ales Ryska, Nils ‘t Hart, Jan H von der Thüsen

**Affiliations:** 1 Department of Public Health and Primary Care, Biomedical Quality Assurance Research Unit, University of Leuven, Leuven, Belgium; 2 Center for Oncologic Research (CORE), University of Antwerp, Antwerp, Belgium; 3 Department of Pathology, University Hospital Antwerp, Edegem, Belgium; 4 Department of Pathology, Charles University Medical Faculty and University Hospital, Hradec Kralove, Czech Republic; 5 Department of Pathology, University Medical Center Groningen, Groningen, The Netherlands; 6 Department of Pathology, Isala Klinieken, Zwolle, The Netherlands; 7 Department of Pathology, University Medical Center Rotterdam, Erasmus MC, Rotterdam, The Netherlands

**Keywords:** PD-L1, Immunohistochemistry, Tumor proportion score, External quality assessment

## Abstract

Programmed death ligand 1 (PD-L1) immunohistochemistry (IHC) is accepted as a predictive biomarker for the selection of immune checkpoint inhibitors. We evaluated the staining quality and estimation of the tumor proportion score (TPS) in non-small-cell lung cancer during two external quality assessment (EQA) schemes by the European Society of Pathology. Participants received two tissue micro-arrays with three (2017) and four (2018) cases for PD-L1 IHC and a positive tonsil control, for staining by their routine protocol. After the participants returned stained slides to the EQA coordination center, three pathologists assessed each slide and awarded an expert staining score from 1 to 5 points based on the staining concordance. Expert scores significantly (*p* < 0.01) improved between EQA schemes from 3.8 (*n* = 67) to 4.3 (*n* = 74) on 5 points. Participants used 32 different protocols: the majority applied the 22C3 (56.7%) (Dako), SP263 (19.1%) (Ventana), and E1L3N (Cell Signaling) (7.1%) clones. Staining artifacts consisted mainly of very weak or weak antigen demonstration (63.0%) or excessive background staining (19.8%). Participants using CE-IVD kits reached a higher score compared with those using laboratory-developed tests (LDTs) (*p* < 0.05), mainly attributed to a better concordance of SP263. The TPS was under- and over-estimated in 20/423 (4.7%) and 24/423 (5.7%) cases, respectively, correlating to a lower expert score. Additional research is needed on the concordance of less common protocols, and on reasons for lower LDT concordance. Laboratories should carefully validate all test methods and regularly verify their performance. EQA participation should focus on both staining concordance and interpretation of PD-L1 IHC.

**Supplementary Information:**

The online version contains supplementary material available at 10.1007/s00428-020-02976-5.

## Introduction

Several immune-checkpoint inhibitors (ICIs) have emerged which target the programmed cell death protein 1 (PD-1)/programmed death ligand 1 (PD-L1) interaction in non-small-cell lung cancer (NSCLC), such as the anti-PD-1 drugs nivolumab and pembrolizumab [1–4], and the PD-L1 inhibitors atezolizumab and durvalumab [5, 6]. The efficacy of ICIs in NSCLC has been shown in various clinical trials, and PD-L1 immunohistochemistry (IHC) has been widely accepted as a predictive biomarker because of its association with increased efficacy of ICIs [7, 8]. Both nivolumab and atezolizumab have been approved by the Food and Drug Administration (FDA) and the European Medicines Agency (EMA) [9, 10] as second-line therapy irrespective of PD-L1 expression. Treatment with pembrolizumab requires at least 50% of PD-L1 positive tumor cells in a first-line setting for stage IV NSCLC patients or those with stage III disease who cannot be treated by chemotherapy or radiation therapy [3, 4]. Recently, the FDA approved durvalumab as maintenance therapy in patients with unresectable stage III NSCLC without progression after concurrent chemoradiotherapy [11], irrespective of the PD-L1 status. The EMA, however, has restricted this indication to patients with PD-L1 on ≥ 1% of tumor cells [12].

Four commercial assays are currently available, each for a specific drug and applying a specific tumor proportion score (TPS) threshold for positivity. The Ventana PD-L1 (SP142) Assay, Ventana PD-L1 (SP263) Assay, and the PD-L1 IHC 22C3 pharmDx (Agilent Technologies/Dako) kit are CE-marked in vitro diagnostic (CE-IVD) labeled [13] and have been validated in the clinical trials for atezolizumab, pembrolizumab or durvalumab, and pembrolizumab only, respectively. The 22C3 kit and SP263 assay have also been approved as companion diagnostics (CDx) for pembrolizumab by the FDA [14] and received a CE-IVD certification in Europe [13], respectively, while the other kits are considered as complementary diagnostics [15].

Several studies have compared these assays, and reported similar analytical sensitivities of the 22C3, 28-8, and SP263 assays with good inter-observer concordance for TPS, but highlighted a lower sensitivity of the SP142 assay in this context [16–22]. In another study, 14/38 (37%) of cases received another clinical classification of the PD-L1 status depending on which assay/scoring system was used when comparing 22C3, 28-8, SP142, and SP263 [18].

Due to the wide variety in commercially available platforms, their concomitant implementation in one laboratory would result in increased costs and a limited number of NSCLC being tested on more than one platform. Laboratories may also opt to use a laboratory developed test (LDT), such as E1L3N or QR1 primary antibodies, or the use of the antibodies described above with a different protocol than the CE-IVD certified ones. Comparison of LDTs to reference CE-IVD assays yielded varying results ranging from 52% to 54% concordance to even 85% or 100 % [17, 23]. To date, however, there remains confusion about the range of assays which are fit-for-purpose for PD-L1 testing for individual drugs and the interchangeability between them.

Irrespective of the protocol used, laboratories are required to appropriately verify or validate their PD-L1 IHC test, to take part in continuous quality monitoring and participation to External Quality Assessment (EQA) [24–26]. Lower staining concordance for LDTs compared with CE-IVD approved assays was reported by two other EQA providers [27–29], but participants’ interpretation of the TPS was not always assessed [27]. The aim of this study is to evaluate the results of assessment of the staining concordance of PD-L1 IHC and its influence on TPS estimations, for the different (LDT or CDx approved) methods in two subsequent EQA schemes of the European Society of Pathology (ESP).

Laboratory characteristics have shown to affect the EQA performance for other markers in NSCLC [30], but not yet for the technical assessment of PD-L1 concordance with optimal reference stains. Therefore, we also aimed to evaluate how different laboratory characteristics influence concordance rates. Finally, we provide an overview of most common staining artifacts observed for our EQA participants.

## Material and methods

Two EQA schemes were organized in 2017 (pilot) and 2018, both accredited for International Organization for Standardization (ISO) 17043:2010 [31] and open to all laboratories worldwide. Participants received two unstained formalin-fixed paraffin embedded (FFPE) slides of 3-μm thickness from a tissue micro-array (TMA) containing three (2017) and four (2018) cases from archival FFPE NSCLC resection specimens (collected 7.4–76.4 months prior to distribution) and a positive tonsil control. In 2017, one large core per case was provided. In 2018, three cores with a diameter of 2 mm were punched for every case. Any one or a combination of the three cores per case could be used for interpretation of the TPS. Hematoxylin and eosin stained slides of parallel sections were made digitally available to enable assessment of tissue morphology, preservation, and the minimum number of tumor cells. To select a sample set with varying TPS and determine the ground truth, samples were pretested by a central accredited reference laboratory [26] with 22C3 (Dako) or SP263 (Ventana) according to manufacturer’s instructions (Supplemental Figure 1).

Participants were requested to stain the slides according to their routine protocol within 14 calendar days after sample receipt and to send the stained slides back to the EQA provider. The maximum time between cutting of the slides and staining by the participants was 1 month. An electronic datasheet was completed including information on the laboratory characteristics, applied methodology, and estimation of the TPS (in categories of < 1%, 1–50%, or > 50%).

A team of three pathologists assessed the stains simultaneously under a multi-head microscope for the staining concordance, based on pre-defined scoring criteria. Prior harmonization was performed for equal assessment on slides with an excellent concordance with the reference stain for a specific antibody. Each participant stain was compared with the optimal reference stain and relative to stains from international peers. An expert staining score (ESS) ranging from 1 to 5 points was awarded based on the staining concordance of all cases with the reference slides, corresponding to 5: Excellent concordance for the specific protocol, 4: Concordant staining with minor remark, 3: Non-concordant staining without affecting clinical output, 2: Non-concordant staining affecting clinical output, 1: Failed, uninterpretable staining.

At the end of the scheme, participants received online examples of optimally concordant stains and corresponding protocols, a general scheme summary on sample outcomes (TPS) and ESS, and individual comments on their individual staining concordances (supplemental Table 1).

In 2018, one of the four cases was excluded, as varying TPS values were reported and no consensus outcome was reached. Thus, six cases were included, two for every TPS category. The reported laboratory settings and accreditation statuses were validated on the websites of the laboratories and their relevant national accreditation bodies [30]. Statistics were performed using SAS software (version 9.4 of the SAS System for Windows, SAS Institute Inc., Cary, NC, USA).The relationship of the ESS on 5 points with laboratory characteristics or used protocols was determined by proportional odds models, presented as odds ratios (OR) with 95% confidence intervals (CIs). The incidence of analysis failures and incorrect TPS estimations related to the ESS and laboratory characteristics was assessed by Poisson models with incidence rate ratios (IRR) with 95% CIs, with the log of the number of EQA samples as an offset variable. Generalized estimating equations (GEE) accounted for clustering in the data (i.e., tests performed by the same laboratory). ‘Approved methods’ were defined as CE-IVD-labeled FDA-approved CDx or complementary diagnostics without a change of protocol. The number of EQA participations, samples tested annually, or involved staff members were considered as ordinal variables (instead of categorical) to evaluate the influence of a +1 level increase.

## Results

In 2017 and 2018, 67 and 74 laboratories participated respectively, resulting in 141 EQA participations from 104 unique laboratories in 30 different countries. The average ESS significantly (*p* < 0.01) improved between 2017 and 2018 from 3.8 to 4.3 points (Table 1); however, there was no significant difference (*p* = 0.2859) between laboratories who participated for the first (4.0) or second (4.2) time.

**Table 1:** Laboratory characteristics related to average PD-L1 IHC ESS, analysis failures, and TPS misclassifications

Characteristic	# observations (%) (*n* = 141)	Average ESS on 5 points^+^	# analysis failures (%) (*n* = 8)^++,°^	# under-estimations (%) (*n* = 20)^++,°^	# over-estimations (%) (*n* = 24)^++,°^
EQA scheme year		0.393 (0.217; 0.713); *p* < 0.01**	ND	0.388 (0.148; 1.015); *p* = 0.0537	0.647 (0.304; 1.377); *p* = 0.2584
2017	67 (47.5)	3.8	0 (0.0)	14 (70.0)	14 (58.3)
2018	74 (52.5)	4.3	8 (100.0)	6 (30.0)	10 (41.7)
# EQA participations		1.430 (0.741; 2.759); *p* = 0.2859	0.937 (0.163; 5.389); *p* = 0.9418	ND	0.402 (0.135; 1.190); *p* = 0.0998
1st participation	104 (73.8)	4.0	6 (75.0)	20 (100.0)	21 (87.5)
2nd participation	37 (26.2)	4.2	2 (25.0)	0 (0.0)	3 (12.5)
Laboratory setting†,‡		*p* = 0.8140	ND	ND	ND
Industry	4 (2.8)	4.3	0 (0.0)	0 (0.0)	3 (12.5)
(private) laboratories	31 (22.0)	4.0	6 (75.0)	4 (20.0)	5 (20.8)
Hospital laboratories	36 (25.5)	4.0	0 (0.0)	8 (40.0)	6 (25.0)
University and research	70 (49.6)	4.1	2 (25.0)	8 (40.0)	10 (41.7)
Accreditation status‡		0.609 (0.313; 1.185); *p* = 0.1442	0.805 (0.133; 4.876); *p* = 0.8136	1.242 (0.492; 3.135); *p* = 0.6466	2.481 (1.049; 5.882); *p* = 0.0386*
No	62 (44.0)	3.8	4 (50.0)	10 (50.0)	16 (66.7)
Yes	77 (54.6)	4.2	4 (50.0)	10 (50.0)	8 (33.3)
Missing data	2 (1.4)	4.5	0 (0.0)	0 (0.0)	0 (0.0)
# samples tested in last 12 months for PD-L1		1.214 (0.874; 1.687); *p* = 0.2475	0.390 (0.150; 1.013); *p* = 0.0532	0.667 (0.426; 1.046); *p* = 0.0779	0.882 (0.591; 1.315); *p* = 0.5376
No clinical testing	7 (5.0)	4.1	3 (37.5)	2 (10.0)	2 (8.3)
< 10	7 (5.0)	3.6	1 (12.5)	4 (20.0)	0 (0.0)
10-99	63 (44.7)	3.8	3 (37.5)	8 (40.0)	11 (45.8)
100-249	32 (22.7)	4.4	0 (0.0)	3 (15.0)	5 (20.8)
250-499	21 (14.9)	4.4	1 (12.5)	3 (15.0)	4 (16.7)
> 500	9 (6.4)	4.0	0 (0.0)	0 (0.0)	0 (0.0)
Missing data	2 (1.4)	4.5	0 (0.0)	0 (0.0)	2 (8.3)
# staff involved in testing		1.212 (0.862; 1.704); *p* = 0.2684	0.637 (0.339; 1.196); *p* = 0.1606	1.123 (0.701; 1.800); *p* = 0.6294	0.970 (0.643; 1.461); *p* = 0.8824
1-5	53 (37.6)	4.0	3 (37.5)	6 (30.0)	8 (33.3)
6-10	49 (34.8)	4.0	5 (62.5)	8 (40.0)	9 (37.5)
11-20	23 (16.3)	4.1	0 (0.0)	4 (20.0)	5 (20.8)
> 20	14 (9.9)	4.4	0 (0.0)	2 (10.0)	1 (4.2)
Missing data	2 (1.4)	5.0	0 (0.0)	0 (0.0)	1 (4.2)
Method type^§^		1.916 (1.012; 3.629); *p* < 0.05	2.716 (0.467; 15.793); *p* = 0.2659	1.350 (0.532; 3.425); *p* = 0.5276	0.789 (0.327; 1.905); *p* = 0.5984
Approved kit (CDx)	67 (47.5)	4.2	2 (25.0)	11 (55.0)	10 (41.7)
LDT	74 (52.5)	3.9	6 (75.0)	9 (45.0)	14 (58.3)
Switched protocol between schemes^¶^		0.899 (0.247; 3.280); *p* = 0.8723	2.083 (0.142; 30.537); *p* = 0.5921	ND	ND
No	25 (17.7)	4.2	1 (12.5)	0 (0.0)	3 (12.5)
Yes	12 (8.5)	4.3	1 (12.5)	0 (0.0)	0 (0.0)
NA	104 (73.8)	4.0	6 (75.0)	20 (100.0)	21 (87.5)
Antibody dilution		*p* < 0.01**	ND	ND	ND
< 1/50	17 (12.1)	4.3	0 (0.0)	1 (5.0)	2 (8.3)
1/50 - 1/100	72 (51.1)	3.9	7 (87.5)	9 (45.0)	12 (50.0)
> 1/100	14 (9.9)	3.4	0 (0.0)	4 (20.0)	4 (16.7)
RTU	38 (27.0)	4.5	1 (12.5)	6 (30.0)	6 (25.0)
Incubation temperature (°C)		0.363 (0.193; 0.682); *p* < 0.01**	ND	ND	ND
RT	55 (39.0)	3.7	1 (12.5)	10 (50.0)	14 (58.3)
30-37	86 (61.0)	4.3	7 (87.5)	10 (50.0)	10 (41.7)
Incubation time (min)		*p* = 0.3784	ND	ND	ND
13-30	78 (55.3)	4.1	3 (37.5)	13 (65.0)	14 (58.3)
31-60	48 (34.0)	4.0	5 (62.5)	6 (30.0)	8 (33.3)
> 60	15 (10.6)	3.9	0 (0.0)	6 (30.0)	2 (8.3)
Use of amplification		1.249 (0.659; 2.365); *p* = 0.4951	ND	ND	ND
No	77 (54.6)	4.1	6 (75.0)	10 (50.0)	12 (50.0)
Yes	64 (45.4)	4.0	2 (25.0)	10 (50.0)	12 (50.0)

*Abbreviations*: # number, *CDx* companion diagnostic, *CI* confidence interval, *EQA* external quality assessment, *ESS* expert staining score, *GEE* generalized estimating equations, *IHC* immunohistochemistry, *IRR* incidence rate ratio, *LDT* laboratory-developed test, *NA* not applicable, *ND* not determined, *OR* odds ratio, *PD-L1* programmed death ligand 1, *RT* room temperature, *RTU* ready-to-use, *TPS* tumor proportion score

+Proportional odds models were used to analyze the difference in ESS. ++Poisson models were used to evaluate the association with analysis failures or under-/over-estimations. Both models applied GEE for clustering of the data. Results are presented as ORs/IRRs (± 95% CI), respectively. OR/IRR > 1 represent a higher ESS/higher incidence for a higher category level. OR/IRR < 1 represent a lower ESS/lower incidence for a higher category level. *p < .05, **p < .01, ***p < .001, ****p < .0001. ND; statistics not computed due to low power (absence or very few events in one level). For variables with more than two categories (laboratory setting, incubation time, and temperature), overall significance levels are given. ORs for every pairwise comparison between categories are described in the main text

°Analysis failures are defined as the failure to stain or interpret the PD-L1 IHC results on all assessed cases. Under-estimations are calculated on samples validated as a TPS of 1–50% or > 50%. Over-estimations are calculated on the total number of samples with TPS < 1% or 1–50%

†Industry are laboratories involved in the development of diagnostic commercial kits. (Private) Laboratories are not within a hospital’s infrastructure. Hospital laboratories included private and public hospitals. University and research included education and research hospitals, university hospitals, university laboratories, and anti-cancer centers [30]

‡Laboratory setting and accreditation were validated on the websites of the laboratories and national accreditation bodies. Accreditation is defined as compliant to ISO 15189 or relevant national standards

§Approved kits are defined as using the Dako 22C3, Ventana SP142, or Ventana SP263 kits with platform for their intended use. LDTs are defined as these three clones in combination with another platform, or any other antibody clone

¶A switch included either the change in primary antibody, antigen retrieval, or detection method. ‘Not applicable’ included entries from first participations for which no method information from previous years was available

Almost half of the 141 participants (49.6%) were university and research (such as specialized cancer centers) laboratories, compared with 25.5% of laboratories affiliated to a general hospital, 22.0% private laboratories, and 2.8% industry laboratories. More than half (54.6%) were accredited for PD-L1 IHC specifically or on a laboratory level according to ISO 15189 or relevant national standards (e.g., College of American Pathologists 15189). The majority of laboratories (63/141, 44.7%) tested on average between 10 and 100 routine clinical samples annually for PD-L1, whereas seven participants (5.0%) did not perform clinical testing. Between 1 and 5 (37.6%) or 6 and 10 (34.8%) staff members were most frequently involved in performing and interpreting the PD-L1 IHC test. The abovementioned laboratory characteristics did not correlate with the ESS in both EQA schemes (Table 1).

The participants stained and interpreted 423 cases in total, of which 371 (87.7%) were correct (i.e., reported TPS was in line with the pre-validated consensus value). In 8 (1.9%) cases, an analysis failure occurred, meaning that the staining could not be performed or interpreted. The TPS was under- and over-estimated in 20 (4.7%) and 24 (5.7%) cases, respectively. The majority of under-estimations occurred for a TPS between 1% and 50%, close to the cutoff value of 1%, while over-estimations where more evenly distributed across TPS categories (Supplemental Table 1).

**Fig. 1:**
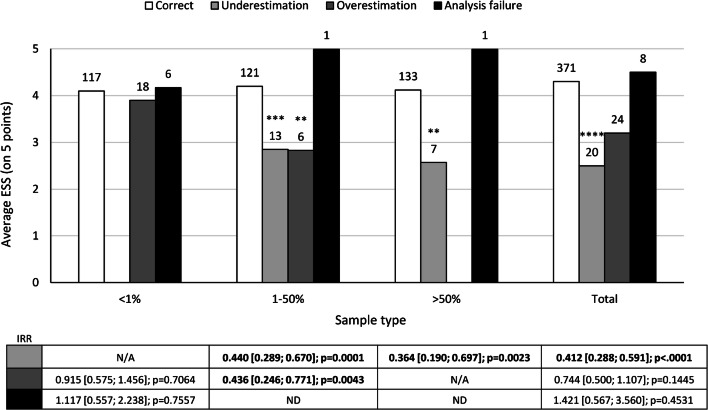
Incidence of analysis failures and TPS under-/over-estimations related to the obtained ESS. Poisson models with GEE were used to analyze the association of the ESS with the number of incorrect TPS classifications (under- or over-estimations) and the number of analysis failures observed in the EQA schemes as count outcome variables. Analysis failures are defined as the failure to stain or interpret the PD-L1 IHC results. Under-estimations are defined only for samples validated as a TPS of 1–50% or > 50%. Over-estimations are defined only for samples with TPS < 1% or 1–50%. Results are presented as IRR (95% CI) taking into account the log of the total number of samples analyzed during the EQA scheme as an offset variable. Bar labels represent the number of cases with correct results/under-estimations/over-estimations/analysis failures observed. IRRs < 1 represent a lower number of incidents for higher ESS. IRRs > 1 represent a higher number of incidents for higher ESS. *p < .05, **p < .01, ***p < .001, ****p < .0001. The IRR for analysis failures in cases with a TPS of 1–50% and > 50% was not computed as only one incident occurred. Abbreviations: CI confidence interval, EQA external quality assessment, ESS expert staining score, GEE generalized estimating equations, IRR incidence rate ratio, N/A not applicable, ND not determined, PD-L1 programmed death ligand 1, TPS tumor proportion score

A lower ESS correlated with TPS under-estimations (*p* < 0.0001) in all cases and over-estimations (*p* < 0.0043) for cases with a TPS between 1% and 50% (Fig. 1). Accredited laboratories less frequently over-estimated cases (*p* < 0.05) (Table 1), but there was no effect on under-estimations. There was no relationship between other laboratory characteristic and incorrect estimations.

For the 8 observed analysis failures, 3 were caused by 1 laboratory unable to interpret the cases as they were still validating their protocol. Another 4 laboratories incorrectly indicated that there were no tumor cells in the provided samples, and 1 participant that their control stained negatively (Supplemental Table 1). The relationship between failures and ESS could not be calculated for cases with a TPS of 1%–50% and > 50% as only 1 failure was observed.

**Fig. 2:**
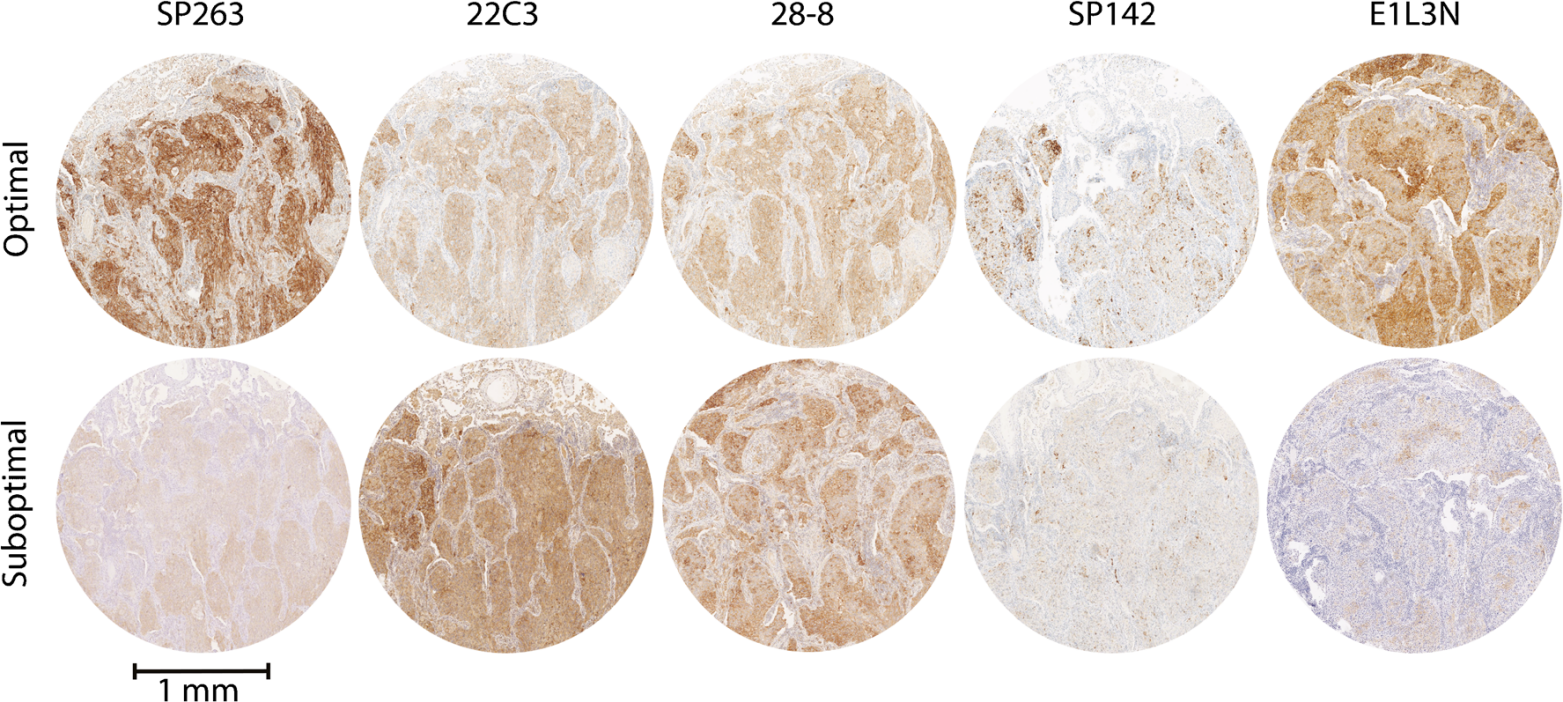
Examples of optimal and suboptimal concordance with PD-L1 IHC reference stains for different protocols during the 2018 EQA scheme. Images represent a matching core with a validated consensus TPS of > 50%. The core was part of a tissue micro-array containing four different FFPE cases for staining by routine antibodies and detection systems of the 2018 EQA participants. Protocols are presented as reported by the participating laboratories. Scale bar 1 mm. Optimal concordance (top row, from left to right): SP263: RTU antibody from Ventana (16 min incubation at 36 °C) in combination with the Ventana CC1 (64 min.) and OptiView DAB IHC Detection Kit. 22C3: Antibody from Dako (diluted 1/40, 16-min incubation at 37 °C) in combination with the Ventana CC1 (64 min) and OptiView DAB IHC Detection Kit. 28-8: Antibody from Abcam (diluted 1/100, 32-min incubation at RT) in combination with the Ventana CC1 (64 min) and OptiView DAB IHC Detection Kit. SP142: RTU antibody from Ventana (24-min incubation at 37 °C) in combination with the Ventana CC1 (48 min) and UltraView DAB IHC Detection Kit. E1L3N: Antibody from Cell Signaling (diluted 1/200, 30-min incubation at RT) in combination with Leica Bond Epitope Retrieval 2 (20 min) and bond polymer refine detection system. Suboptimal concordance (bottom row, from left to right): SP263: RTU antibody from Ventana (60-min incubation at 37 °C) in combination with the Ventana CC1 (64 min) and OptiView DAB IHC Detection Kit; weak demonstration of antigen in the tumor population and cytoplasmic staining. 22C3: Antibody from Dako (diluted 1/50, 30-min incubation at RT) in combination with Dako EnVisionFLEX Target Retrieval Solution (low pH) and the Envision Flex detection system; Excessive background staining and cytoplasmic staining. 28-8: Antibody from Abcam (diluted 1/50, 20-min incubation at 32 °C) in combination with Dako EnVisionFLEX Target Retrieval Solution (low pH) and the Envision Flex detection system; background staining. SP142: RTU antibody from Ventana (16-min incubation at 36 °C) in combination with the Ventana CC1 (48 min) and OptiView DAB IHC Detection Kit; weak staining of epithelial cells. E1L3N: Antibody from Cell Signaling (diluted 1/150, 60-min incubation at RT) in combination with laboratory developed antigen retrieval by TRIS-EDTA and the Vectastain ABC immunoperoxidase staining avidin-biotin complexes; weak demonstration of antigen in the tumor population and cytoplasmic staining. Abbreviations: EQA external quality assessment, FFPE formalin-fixed paraffin embedded, IHC immunohistochemistry, PD-L1 programmed death ligand 1, RT room temperature, RTU ready-to-use, TPS tumor proportion score

In total, 81/141 participants did not obtain the maximum ESS of 5 on 5 points and received individual feedback. The majority of issues observed included a very weak (28.4%) or weak (34.6%) demonstration of the antigen in the tumor population, as well as slight (12.4%) or excessive (7.4%) background staining, not related to the used protocol (Supplemental Table 2). Examples of most frequently observed staining artifacts are given in Fig. 2.

The applied test methods for PD-L1 IHC significantly influenced the ESS. CE-IVD labeled or CDx kits (e.g., Ventana PD L1 (SP142) Assay, Ventana PD L1 (SP263), and the PD-L1 IHC 22C3 pharmDx) reached a higher ESS (4.2/5, *n* = 67) compared with LDTs (3.9/5, *n* = 74) (OR1.916 [1.012; 3.626], *p* < 0.05) (Table 1). To assess if a recent change in protocol negatively affected the ESS, we evaluated the difference in ESS for participants who did or did not change their method between both schemes. Exactly 104 participants (73.8%) were excluded as they were first time participants, and no method information from previous years was available. For the remaining 37 laboratories, 12 changed their test method (either the primary antibody, antigen retrieval, or detection method), but no difference in ESS was observed (OR 0.899 [0.247; 3.280], *p* = 0.8723).

The use of a ready-to use (RTU) antibody dispenser yielded significantly higher ESS compared with using a specific dilution factor between 1/50 and 1/100 (OR 5.025 [2.058; 12.346], *p* = 0.0004) or > 1/100 (OR 9.009 [2.169; 37.037]; *p* = 0.0024), but not compared with a dilution factor of < 1/50 (OR 2.681 [0.924; 7.752]; *p* = 0.0696) (data not shown). In contrast, incubation at room temperature (RT) reduced the ESS compared with higher temperatures (Table 1). The incubation time or the use of amplification during detection did not alter the ESS. Because of the low frequency of technical failures and misclassifications, their percentages are given on a descriptive level only and no ORs are provided.

In total, the EQA participants used 32 different combinations of primary antibodies, antigen retrieval, and detection methods (Table 2). Out of 140 participations, the most widely used primary antibody was the 22C3 antibody (Dako) (56.7%), followed by SP263 (Ventana) (19.1%), and E1L3N (Cell Signaling) (7.1%). The remaining 16.8% of participants used less common primary antibodies, namely, 28-8 (Abcam, 2.8%), 28-8 (Dako, 2.8%), CAL10 (Biocare Medical, 2.8%), QR1 (Quartett, 4.9%), and SP142 (Ventana, 3.5%) (Table 2).

**Table 2:** Analysis failures, TPS misclassifications, and ESS for the different PD-L1 IHC protocols used in the EQA schemes

Primary antibody	Antigen retrieval	Detection method	# times used (%) (*n* = 141)	# analysis failures (%) (*n* = 8)	# Under-estimations (%) (*n* = 20)	# Over-estimations (%) (*n* = 24)	Average ESS/5	Method code	OR (95% CI) relative to method					
									a	b	c	d	e	f
22C3 (Dako)	Cc1 (Ventana)	OptiView DAB IHC Detection Kit (Ventana)	35 (24.8%)	5 (62.5%)	3 (15.0%)	3 (12.5%)	4.1	a	/	1.247 (0.547; 2.843)	4.075 (1.314; 12.632)*	0.129 (0.042; 0.396)***	1.769 (0.482; 6.494)	1.211 (0.422; 3.481)
	EnVisionFLEX Target Retrieval Solution, low pH (Dako)	Envision flex (Dako)	32 (22.7%)	1 (12.5%)	3 (15.0%)	5 (20.8%)	3.9	b	0.802 (0.352; 1.828)	/	3.269 (0.979; 10.919)	0.103 (0.029; 0.371)***	1.419 (0.361; 5.579)	0.972 (0.311; 3.037)
	Bond Epitope Retrieval 1 (Leica)	Bond polymer refine detection system (Leica)	1 (0.7%)	0 (0.0%)	0 (0.0%)	2 (8.3%)	1.0	c	0.245 (0.079; 0.761)*	0.306(0.092; 1.022)	/	0.032 (0.007; 0,147)****	0.434 (0.090; 2.102)	0.297 (0.075; 1.178)
	Bond Epitope Retrieval 2 (Leica)		2 (1.4%)	1 (12.5%)	0 (0.0%)	1 (4.2%)	3.5							
	Cc1 (Ventana)	UltraView Universal DAB Detection kit (Ventana)	4 (2.8%)	0 (0.0%)	2 (10.0%)	0 (0.0%)	3.3							
	Omnis Envision FLEX TRS, High pH (Dako)	Envision flex (Dako)	1 (0.7%)	0 (0.0%)	2 (10.0%)	0 (0.0%)	2.0							
	PT module TRS High envision Flex (Dako)		2 (1.4%)	0 (0.0%)	0 (0.0%)	0 (0.0%)	4.0							
	Homebrew EDTA or TRIS-EDTA (with/without pressure cooker)	Bond polymer refine detection system (Leica)	1 (0.7%)	0 (0.0%)	0 (0.0%)	0 (0.0%)	3.0							
	Ultra CC1 (Ventana)	OptiView DAB IHC Detection Kit (Ventana)	2 (1.4%)	0 (0.0%)	0 (0.0%)	1 (4.2%)	4.5							
SP263 (Ventana)	Cc1 (Ventana)	OptiView DAB IHC Detection Kit (Ventana)	24 (17.0%)	1 (12.5%)	1 (5.0%)	5 (20.8%)	4.8	d	7.752 (2.525; 23.810)***	9.709 (2.695; 34.483)***	31.660 (6.809; 147.22)****	*/*	13.746 (2.699; 70.007)**	9.412 (2.466; 35.916)**
		UltraView Universal DAB Detection kit (Ventana)	2 (1.4%)	0 (0.0%)	0 (0.0%)	0 (0.0%)	5.0		ND	ND	ND	ND	ND	ND
	EnVisionFLEX Target Retrieval Solution, low pH (Dako)	OptiView DAB IHC Detection Kit (Ventana)	1 (0.7%)	0 (0.0%)	0 (0.0%)	0 (0.0%)	5.0		ND	ND	ND	ND	ND	ND
E1L3N (cell signaling)	Bond Epitope Retrieval 2 (Leica)	Bond polymer refine detection system (Leica)	6 (4.3%)	0 (0.0%)	0 (0.0%)	1 (4.2%)	4.3	e	0.565 (0.154; 2.075)	0.705 (0.179; 2.770)	2.304 (0.476; 11.111)	0.073 (0.014; 0.371)**	/	0.685 (0.149; 3.155)
	Homebrew EDTA or TRIS-EDTA (w/wo pressure cooker)	ABC immunoperoxidase staining avidin-biotin complexes (Vectastain ABC Elite; Vector Laboratories)	1 (0.7%)	0 (0.0%)	1 (5.0%)	0 (0.0%)	1.0							
		Bond polymer refine detection system (Leica)	1 (0.7%)	0 (0.0%)	0 (0.0%)	0 (0.0%)	4.0							
		Brightvision(Immunologic)	1 (0.7%)	0 (0.0%)	0 (0.0%)	0 (0.0%)	3.0							
		ZytoChem Plus (HRP) Polymer Kit (Zytomed)	1 (0.7%)	0 (0.0%)	0 (0.0%)	0 (0.0%)	3.0							
28-8 (Abcam)	Cc1 (Ventana)	OptiView DAB IHC Detection Kit (Ventana)	1 (0.7%)	0 (0.0%)	0 (0.0%)	0 (0.0%)	5.0	f	0.826 (0.287; 2.370)	1.029 (0.329; 3.215)	3.364 (0.849; 13.321)	0.106 (0.028; 0.406)**	1.461 (0.317; 6.719)	/
	DAKO Omnis Envision FLEX TRS, High pH	Envision flex (Dako)	2 (1.4%)	0 (0.0%)	0 (0.0%)	0 (0.0%)	3.5							
	EnVisionFLEX Target Retrieval Solution, low pH (Dako)		1 (0.7%)	0 (0.0%)	0 (0.0%)	0 (0.0%)	4.0							
28-8 (Dako)	Cc1 (Ventana)	OptiView DAB IHC Detection Kit (Ventana)	1 (0.7%)	0 (0.0%)	0 (0.0%)	0 (0.0%)	4.0							
	EnVisionFLEX Target Retrieval Solution, low pH (Dako)	Envision flex (Dako)	3 (2.1%)	0 (0.0%)	0 (0.0%)	0 (0.0%)	4.7							
CAL10 (Biocare Medical)	Bond Epitope Retrieval 2 (Leica)	Bond polymer refine detection system (Leica)	1 (0.7%)	0 (0.0%)	0 (0.0%)	0 (0.0%)	4.0							
	Cc1 (Ventana)	OptiView DAB IHC Detection Kit (Ventana)	1 (0.7%)	0 (0.0%)	0 (0.0%)	1 (4.2%)	2.0							
	Homebrew EDTA or TRIS-EDTA (with/without pressure cooker)	Master Polymer Plus (Master Diagnóstica SLU)	1 (0.7%)	0 (0.0%)	0 (0.0%)	1 (4.2%)	5.0							
		ZytoChem Plus (HRP) Polymer Kit (Zytomed)	1 (0.7%)	0 (0.0%)	0 (0.0%)	2 (8.3%)	2.0							
QR1 (Quartett)	Bond Epitope Retrieval 1 (Leica)	Bond polymer refine detection system (Leica)	2 (1.4%)	0 (0.0%)	1 (5.0%)	0 (0.0%)	2.0							
	Cc1 (Ventana)	UltraView Universal DAB Detection kit (Ventana)	3 (2.1%)	0 (0.0%)	0 (0.0%)	0 (0.0%)	5.0							
	EnVisionFLEX Target Retrieval Solution, low pH (Dako)	Envision flex (Dako)	1 (0.7%)	0 (0.0%)	1 (5.0%)	1 (4.2%)	5.0							
	Homebrew EDTA or TRIS-EDTA (with/without pressure cooker)	ZytoChem Plus (HRP) Polymer Kit (Zytomed)	1 (0.7%)	0 (0.0%)	1 (5.0%)	1 (4.2%)	4.0							
SP142 (Ventana)	Cc1 (Ventana)	OptiView DAB IHC Detection Kit (Ventana)	4 (2.8%)	0 (0.0%)	4 (20.0%)	0 (0.0%)	2.8							
		UltraView Universal DAB Detection kit (Ventana)	1 (0.7%)	0 (0.0%)	1 (5.0%)	0 (0.0%)	5.0							

Proportional odds models with GEE for clustering of the data were used to analyze the difference in ESS. Differences in ESS are represented as ORs (95% CI) for every method (row level) relative to other methods used (column level). OR > 1 represent a higher ESS for a given method (column level) relative to the other method (row level). OR < 1 represent a lower ESS for a method relative to other methods. Statistics are computed for main method categories. ND; statistics not computed due to low power (low number of users). Significant results are highlighted in italics. *p < .05, **p < .01, ***p < .001, ****p < .0001. Analysis failures are defined as the failure to stain or interpret the PD-L1 IHC results on all assessed cases. Under-estimations are calculated on samples validated as a TPS of 1–50% or > 50%. Over-estimations are calculated on the total number of samples with TPS < 1% or 1–50%

*Abbreviations*: # number, *CI* confidence interval, *EQA* external quality assessment, *ESS* expert staining score, *GEE* generalized estimating equations, *IHC* immunohistochemistry, *ND* not determined, *OR* odds ratio, *PD-L1* programmed death ligand 1, *TPS* tumor proportion score

We compared the most frequently used protocols with other protocols (Table 2). The SP263 (Ventana) CDx kit (with the Cc1 antigen retrieval and OptiView DAB IHC Detection Kit) displayed significantly higher ESS compared with all other protocols (LDTs and approved kits) (Table 2, code d). The most frequently used antibody, 22C3, yielded varying ESS depending on the detection platform used. For instance, 22C3 in combination with less commonly used antigen retrieval and detection methods (not included in the CDx kit) (Table 2, code c) resulted in significantly lower ESS compared with 22C3 with reagents from the CDx kit (EnVisionFLEX Target Retrieval Solution and Envision Flex detection method), or with the Optiview platform. We observed no other statistical differences in ESS for the other methods.

## Discussion

Detection of PD-L1 expression is a valuable biomarker in NSCLC to select patients for ICI treatment [8]. Many studies have emphasized the variation in techniques, positivity thresholds, and staining concordance [15,23, 25, ].

This study for the first time correlated the ESS with the different protocols, laboratories’ characteristics, and the incidence of reporting an incorrect TPS.

First, our results confirm a wide variety of testing protocols used across Europe not only for the primary antibodies but also for the different detection methods, with an overall better staining concordance for FDA Cdx approved kits, compared with LDTs.

It must be noted that the number of users in this study could have contributed to the difference between CE-IVD kits and LDTs, as the SP263 and 22C3 assays made up 17.0% and 22.7% (Table 2) of performed tests. Some antibody clones, such as SP142 or other LDTs, had only a limited number of users, and results should be interpreted with caution. The same is true for other non-commercial antibodies reported in literature with a lower sensitivity than E3L1N, such as the 5H1, 7G11, 015, and 9A11 [32, 33], which were not assessed due to an absence of users in these EQA schemes.

An explanation for better concordance of CE-IVD marked kits might also include (i) the reduced inter-laboratory variation by restricting of the protocol in automatic software deployers, (ii) the associated chemistry used to build these assays [32], or (iii) difficulties in adhering to existing validation guidelines [25, 26, 34] for LDTs, as a gold standard for PD-L1 assays and cut-offs is not available [33]. Additional research into the different validation practices of the participants might provide a better insight as to why LDTs are currently underperforming. Some previous studies confirm our results, in which fewer LDTs passed the quality control compared with the clinically validated assays for PD-L1 [17, 27–29], and for ALK receptor tyrosine kinase IHC [35, 36]. However, other studies reported a high concordance of LDTs with reference assays [21, 37]. With the change of the CE-IVD directive into a European IVD regulation, more stringent validations need to be performed for the kits to retain their label, possibly resulting in more laboratories switching to approved kits [13, 38]. Continued data are thus needed to confirm the lower concordance of LDTs in these EQA schemes.

Even though we compared the broad categories of LDTs versus CE-IVD kits, we also observed variability within each category, demonstrated by the higher concordance of the SP263 CE-IVD kit compared with the 22C3 CDx kit, but also the high concordance of the E1L3N primary antibody (Cell Signaling) compared with other LDTs. This is in line with previously reported results, both for E1L3N [17] and for SP263 [39].

Secondly, we observed an effect of antibody dilution and incubation temperature, with higher concordance for RTU antibody dilutions (compared with a dilution factor between 1/50 and 1/100) and for incubation between 30 °C and 37 °C (compared with RT). However, that might be explained as the majority of the data were derived from RTU antibodies as part of CDx commercial kits. Although amplification has been reported to alter the test outcome for expression levels near a cut-off [40], we did not observe a difference.

Third, under- or over-estimations should be avoided, as they could significantly alter the treatment options for patients. In this study, their absolute frequency was low, and laboratories overall interpreted the PD-L1 IHC outcomes well, especially given that PD-L1 is a relatively novel marker and increased error rates were reported during the introduction of novel markers into practice [18, 30]. The TPS estimation was however significantly affected by the ESS, resulting in under-estimations for lower ESS. This emphasizes that rather than interpretation of the obtained staining pattern, key to a correct result is selecting an appropriate staining protocol with careful validation and quality monitoring. Moreover, it is important that both laboratories and EQA assessors calibrate the outcome for each staining protocol with respect to the optimal staining for that specific antibody.

The majority of misclassifications occurred at the threshold cut-off, which is a well-known problem [39], mainly due to weak demonstration of the antigen in the tumor population or excessive background staining (Supplemental table 2), resulting in the loss of the signal to background ratio. Even TPS values differing by 20% or more compared with the validated outcome were observed (Supplemental table 1). Therefore, sample switches (e.g., confusion about which core belongs to which case on the TMA) cannot be excluded.

In contrast to under-estimations, there was no significant relationship between the ESS and analysis failures, and overall incidence of these failures was low. While 4 laboratories indicated a lack of neoplastic cells in the sample, this could not be confirmed by microscopic review of the slides by the assessors, and peers who received slide sets from a similar position in the tissue block did not report any problems.

It must be noted that the schemes were performed on TMAs with 1 or 3 cores per case, and might not completely reflect the entire tumor microenvironment or PD-L1 expression on the invasive tumor front seen in routine practice [41]. Nevertheless, EQA results from the participants were correlated to the received tissue section, and cases displaying heterogeneity were excluded from the concordance assessment (Supplemental Figure 1).

Fourth, this is the first study to evaluate the relationship between different laboratory characteristics and the ESS. We observed a significant improvement over time from 3.8 to 4.3 on 5 points (*p* < 0.01). Even though second-time participants had a higher ESS and fewer incorrect outcomes/analysis failures, results were not significant. It must be noted that only 37 laboratories participated in both schemes and the TMAs sent in 2017 and 2018 were different (Supplemental Figure 1). More longitudinal data are needed to confirm a positive effect of repeated EQA participation and the feedback provided, as previously suggested [42].

Interestingly, while accreditation was significantly associated with fewer misclassifications, this was not the case for the ESS. Even when using an optimal IHC protocol, interpretation of the PD-L1 status might still be subjective based on the correct separation of membrane staining of the neoplastic cells versus non-neoplastic epithelial cells, immune cells, and necrosis.

The fact that laboratory accreditation affected the interpretation, but not the staining concordance, suggests that laboratories should participate in both aspects. From the EQA providers’ side, schemes should be fit-for-purpose to assess both staining concordance and interpretation [43, 44]. Previously, interpretation of PD-L1 IHC results has been described to improve upon pathologist training [17]. In our schemes, PD-L1 IHC was more frequently performed in research institutes, but laboratory setting and experience (number of samples tested, number of staff members involved, and change in methodology) did not correlate with overall ESS or TPS interpretation, in contrast to previously reported data [30]. As we included data from only two subsequent EQA schemes, additional schemes might bring more clarity on the effect of laboratory characteristics.

To conclude, the increasingly complex testing paradigm for PD-L1 poses many challenges for pathologists and oncologists. EQA participation could guide laboratories in obtaining better concordance. The use of a CE-IVD kit according to the manufacturer’s instructions positively influences EQA concordance, even though additional research is needed on less common protocols and non-automated techniques. In addition, EQA participation should include a technical evaluation, given that lower ESS was shown to be at the basis of TPS misclassifications, rather than interpretation issues, and both aspects were differently affected by the laboratory characteristics.

One of the advantages of the EQA schemes is the large participants group for which a TPS estimation is available, allowing to determine an optimal consensus TPS for every case, and objectively comparing protocols by eliminating influences of the pre-analytical phase (i.e., all participants receiving similar and pre-validated slides). It remains to be elucidated how these findings are reflected in routine settings, where different pre-analytical variables and sampling of heterogeneous biopsies can occur. Additionally, supplementing research on the errors made (e.g., personnel errors when following the protocol, clerical errors when reporting the outcomes) might reveal shortcomings in individual laboratories leading to lower concordance in the EQA scheme.

## Supplementary information

ESM 1 (DOCX 128 kb)

ESM 2 (DOCX 17 kb)

ESM 3 (DOCX 21 kb)

## Data Availability

The datasets generated during and/or analyzed during the current study are available from the corresponding author on reasonable request.

## Abbreviations

CI: Confidence interval
CDx: Companion diagnostic
EQA: External quality assessment
ESS: Expert staining score
FDA: Food and Drug Administration
FFPE: Formalin-fixed paraffin embedded
EGFR: Epidermal growth factor receptor
EMA: European Medicines Agency
ESP: European Society of Pathology
GEE: Generalized estimating equations
IHC: Immunohistochemistry
ICI: Immune Checkpoint Inhibitors
IRR: Incidence rate ratio
ISO: International Organization for Standardization
NSCLC: Non-small-cell lung cancer
OR: Odds ratio
PD-1: Programmed cell death protein 1
PD-L1: Programmed death ligand 1
RT: Room temperature
RTU: Ready-to-use
TPS: Tumor proportion score
TMA: Tissue micro-array
