# Supplementary material for: PD-L1 immunohistochemistry in non-small-cell lung cancer: unraveling differences in staining concordance and interpretation
Source: Virchows Arch. 2020 Dec 4;478(5):827–39. doi: 10.1007/s00428-020-02976-5 (PMC8099807; doi:10.1007/s00428-020-02976-5)
Supplement: Supplementary file 1 — (DOCX 128 kb) [file 428_2020_2976_MOESM1_ESM.docx]

Title **PD-L1 immunohistochemistry in non-small cell lung cancer: unraveling differences in staining concordance and interpretation**

Journal Virchows Archiv

Authors Cleo Keppens, Elisabeth MC Dequeker, Patrick Pauwels, Ales Ryska, Nils ‘t Hart, Jan H von der Thüsen

Correspondence Dr. Jan von der Thüsen

University Medical Centre Rotterdam (Erasmus MC)

Department of Pathology

Dr. Molewaterplein 40

3015 GD Rotterdam

The Netherlands

Tel. +31 (0)10 704 44 25

E-mail: j.vonderthusen@erasmusmc.nl

Resources: **Supplemental Figure 1. Reference stains of the TMA sections provided to the**

**participants.**

**Supplemental Figure 1. Reference stains of the TMA sections provided to the participants.**


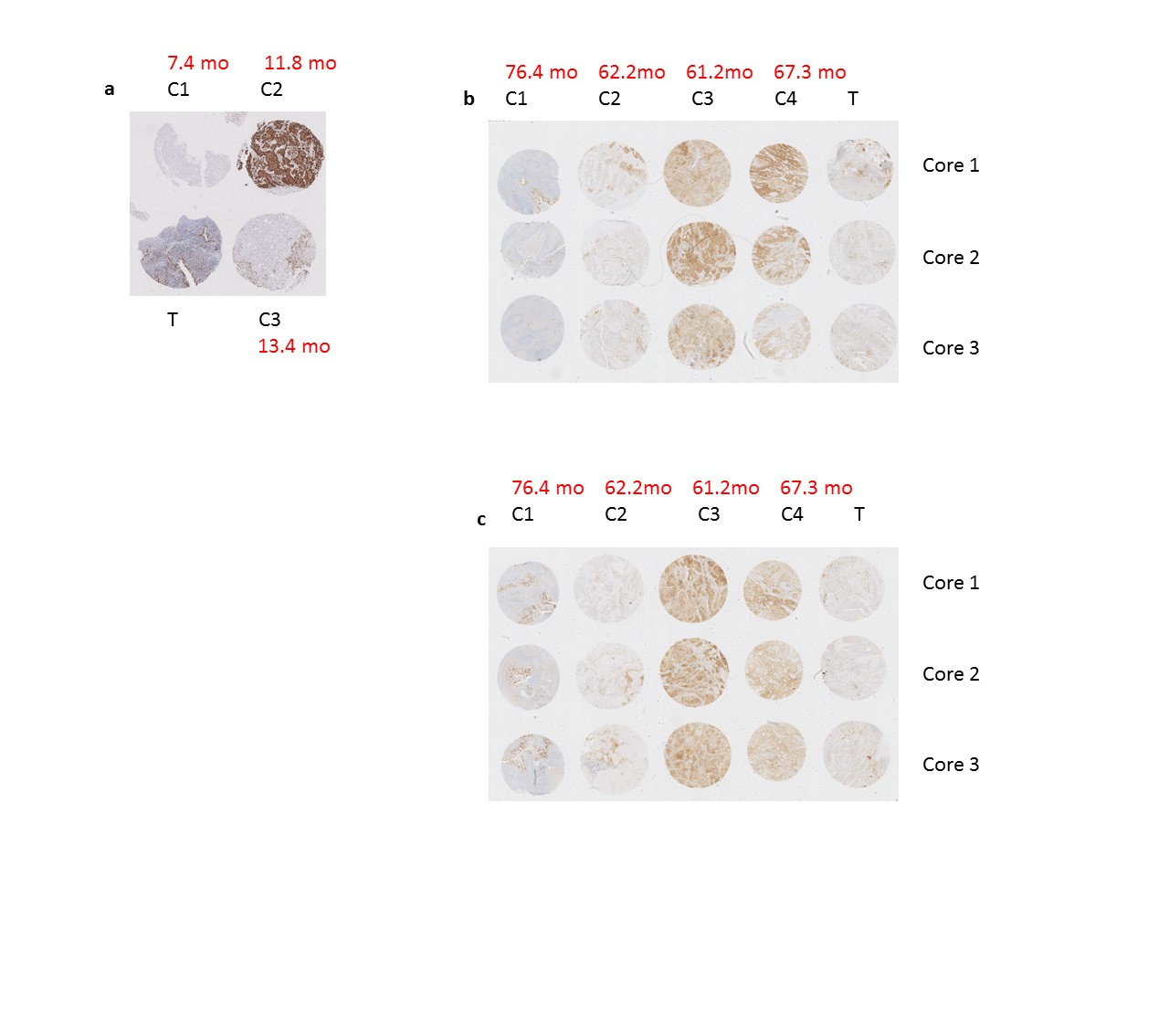


Overview of the reference TMA sections provided to the participants in 2017 (panel a) and 2018 (panels b, c). **Panel a.** TMA block used in 2017. **Panel b.** TMA-block 1 from 2018. **Panel c.** TMA-block 2 from 2018.

In 2017, staining was performed by 22C3 (Dako) and the Optiview kit (Ventana). One large core was provided per case. The pre-validated TPS outcomes in 2017 (**panel a**) were: C1: <1%, C2:>50%, C3:1-50%.

In 2018, staining was performed by SP263 (Ventana) according to the manufacturer’s instructions (using an up-to-date deployer) on a BenchMark Ultra system (Roche Diagnostics). Two different TMA blocks from identical originating material were used to cut a series of slides for the participants. For each case, three smaller cores were provided. The validated outcomes (**panels b, c**) were: C1: <1% or 1-10%, C2: 1-10%, C3: >50%, C4: 1-50%. In 2018, C2 was excluded from the EQA scheme performance scores, as varying TPS values were reported by the participants (despite the initial validation of 1-10%) and no consensus outcome was reached.

In both scheme years, hematoxylin and eosin stains were made every 50 slides to evaluate sufficient tumor content and cell morphology. Red numbers display the age of the used sample material (i.e. time between sample collection and distribution to participants, expressed in months) for every case. Tonsil materials were collected during the same year as the scheme distribution. The maximum time between cutting of the slides and staining by the participants was 1 month. Reported TPS by the participants were correlated with the level of tissue sections in the block to limit the possibility of tissue heterogeneity as a cause for incorrect results. Cases with suspicion of heterogeneity were excluded from the performance calculations.

Abbreviations: C1, Case 1; C2, Case 2; C3, Case 3; C4, Case 4; T, tonsil control; TMA, tissue micro-array; TPS, tumor proportion score.
