# Supplementary material for: PD-L1 immunohistochemistry in non-small-cell lung cancer: unraveling differences in staining concordance and interpretation
Source: Virchows Arch. 2020 Dec 4;478(5):827–39. doi: 10.1007/s00428-020-02976-5 (PMC8099807; doi:10.1007/s00428-020-02976-5)
Supplement: Supplementary file 2 — (DOCX 17 kb) [file 428_2020_2976_MOESM2_ESM.docx]

Title **PD-L1 immunohistochemistry in non-small cell lung cancer: unraveling differences in staining concordance and interpretation**

Journal Virchows Archiv

Authors Cleo Keppens, Elisabeth MC Dequeker, Patrick Pauwels, Ales Ryska, Nils ‘t Hart, Jan H von der Thüsen

Correspondence Dr. Jan von der Thüsen

University Medical Centre Rotterdam (Erasmus MC)

Department of Pathology

Wytemaweg 80 Box 2040

3000 CB, Rotterdam

The Netherlands

Tel. +31 (0)10 704 44 25

E-mail: j.vonderthusen@erasmusmc.nl

Resources: **Supplemental table 1: Overview of detailed TPS estimations in increments of 10% for the different outcomes and reasons for analysis failures observed in the EQA scheme.**

**Supplemental Table 1: Overview of detailed TPS estimations in increments of 10% for the different outcomes and**

**reasons for analysis failures observed in the EQA scheme.**

Abbreviations: #, number; EQA, External Quality Assessment; TPS, tumor proportion score.

|  | # participants | | |
| --- | --- | --- | --- |
| Sample type | <1% (n=141) | 1%-50% (n=141) | >50% (n=141) |
|  | Reported TPS in 10% increments for correct outcomes,   underestimations and overestimations (# of labs) | | |
| <1% | 117 | 13 | 0 |
| 1-10% | 3 | 40 | 2 |
| 11-20% | 9 | 32 | 1 |
| 21-30% | 4 | 30 | 2 |
| 31-40% | 0 | 17 | 2 |
| 41-50% | 0 | 1 | 0 |
| 51-60% | 0 | 2 | 10 |
| 61-70% | 1 | 1 | 14 |
| 71-80% | 0 | 1 | 21 |
| 81-90% | 0 | 0 | 35 |
| 91-100% | 1 | 2 | 53 |
|  | Reasons for analysis failures | | |
| Still validating technique, unable to interpret | 1 | 1 | 1 |
| No staining was observed in the tonsil control | 1 | 0 | 0 |
| No neoplastic cells present in the sample | 3 | 0 | 0 |
| Too few neoplastic cells for the technique used | 1 | 0 | 0 |
