# Supplementary material for: PD-L1 immunohistochemistry in non-small-cell lung cancer: unraveling differences in staining concordance and interpretation
Source: Virchows Arch. 2020 Dec 4;478(5):827–39. doi: 10.1007/s00428-020-02976-5 (PMC8099807; doi:10.1007/s00428-020-02976-5)
Supplement: Supplementary file 3 — (DOCX 21 kb) [file 428_2020_2976_MOESM3_ESM.docx]

Title **PD-L1 immunohistochemistry in non-small cell lung cancer: unraveling differences in staining concordance and interpretation**

Journal Virchows Archiv

Authors Cleo Keppens, Elisabeth MC Dequeker, Patrick Pauwels, Ales Ryska, Nils ‘t Hart, Jan H von der Thüsen

Correspondence Dr. Jan von der Thüsen

University Medical Centre Rotterdam (Erasmus MC)

Dr. Molewaterplein 40

3015 GD, Rotterdam

The Netherlands

Tel. +31 (0)10 704 44 25

E-mail: j.vonderthusen@erasmusmc.nl

Resources: **Supplemental table 2: Individual feedback provided to participants at the end of the EQA scheme related to test methodology.**

**Supplemental Table 2: Individual feedback provided to participants at the end of the EQA scheme related to test methodology.**

Depending on the staining concordance, more than one comment could be provided simultaneously. Numbers between brackets represent the percentage of users receiving the comment for that respective methodology. Abbreviations: EQA, External Quality Assessment.

| **Method** | **# times used in EQA scheme (2017-2018)** | **Very weak staining of control** | **Slightly weaker demonstration of the antigen in the tumor population** | **Weak demonstration of antigen in the tumor population** | **Very weak demonstration antigen in the tumor population** | **Slight background staining** | **Excessive background staining** | **Counter-stain is pale** | **Excessive pre-treatment (enzymatic or heat mediated)** | **Excessive antigen retrieval** | **Inappropriate / non-specific staining of some cells** | **Excessive staining of epithelial cells** | **Cytoplasmatic staining** |
| --- | --- | --- | --- | --- | --- | --- | --- | --- | --- | --- | --- | --- | --- |
| 22C3 (Dako) + OptiView DAB IHC Detection Kit (Ventana) | 37 | 1 (2.7) | 3 (8.1) | 10 (27.0) | 8 (21.6) | 1 (2.7) | 1 (2.7) | 2 (5.4) | 1 (2.7) | 1 (2.7) |  |  |  |
| 22C3 (Dako) + Envision flex (Dako) | 35 | 2 (5.7) | 2 (5.7) | 9 (25.7) | 6 (17.1) |  | 2 (5.7) | 1 (2.9) | 1 (2.9) |  |  |  | 1 (2.9) |
| 22C3 (Dako) + UltraView Universal DAB Detection kit (Ventana) | 4 |  |  | 3 (75.0) | 1 (25.0) |  | 2 (50.0) | 1 (25) |  |  |  |  |  |
| 22C3 (Dako) + Bond polymer refine detection system (Leica) | 4 |  | 1 (25.0) |  | 2 (50.0) |  | 1 (25.0) |  |  |  |  |  |  |
| SP263 (Ventana) + OptiView DAB IHC Detection Kit (Ventana) | 25 |  |  | 3 (12.0) |  |  |  | 1 (4.0) |  |  |  |  | 1 (4.0) |
| 28-8 (Abcam) + Envision flex (Dako) | 3 |  |  |  |  | 1 (33.3) |  |  |  |  |  |  |  |
| 28-8 (Dako) + Envision flex (Dako) | 3 |  |  | 1 (33.3) |  | 1 (33.3) |  |  |  |  |  |  |  |
| 28-8 (Dako) + OptiView DAB IHC Detection Kit (Ventana) | 1 |  |  |  |  | 1 (100.0) |  |  |  |  |  |  |  |
| CAL10 (Biocare Medical) + Bond polymer refine detection system (Leica) | 1 |  |  | 1 (100.0) |  |  |  |  |  |  |  |  |  |
| CAL10 (Biocare Medical) + ZytoChem Plus (HRP) Polymer Kit (Zytomed) | 1 |  |  |  |  |  | 1 (100.0) | 1 (100.0) |  |  | 1 (100.0) |  |  |

| **Method *(Continued)*** | **# times used in EQA scheme (2017-2018)** | **Very weak staining of control** | **Slightly weaker demonstration of the antigen in the tumor population** | **Weak demonstration of antigen in the tumor population** | **Very weak demonstration antigen in the tumor population** | **Slight background staining** | **Excessive background staining** | **Counter-stain is pale** | **Excessive pre-treatment (enzymatic or heat mediated)** | **Excessive antigen retrieval** | **Inappropriate / non-specific staining of some cells** | **Excessive staining of epithelial cells** | **Cytoplasmatic staining** |
| --- | --- | --- | --- | --- | --- | --- | --- | --- | --- | --- | --- | --- | --- |
| E1L3N (cell signaling) + Bond polymer refine detection system (Leica) | 7 |  |  |  | 1 (14.3) | 2 (28.6) | 1 (14.3) |  |  |  |  |  | 1 (14.3) |
| E1L3N (cell signaling) + ABC immunoperoxidase staining avidin-biotin complexes (Vector Laboratories) | 1 |  |  |  | 1 (100.0) |  |  |  |  |  |  |  |  |
| E1L3N (cell signaling) + Brightvision(Immunologic) | 1 |  |  |  |  |  | 1 (100.0) |  |  |  |  |  |  |
| E1L3N (cell signaling) + ZytoChem Plus (HRP) Polymer Kit (Zytomed) | 1 |  |  |  | 1 (100.0) |  | 1 (100.0) |  |  |  |  |  |  |
| QR1 (Quartett) + Bond polymer refine detection system (Leica) | 2 |  |  |  | 2 (100.0) |  |  |  |  |  |  |  |  |
| QR1 (Quartett) + ZytoChem Plus (HRP) Polymer Kit (Zytomed) | 1 |  |  | 1 (100.0) |  |  |  |  |  |  |  |  |  |
| SP142 (Ventana) + OptiView DAB IHC Detection Kit (Ventana) | 4 |  |  |  | 1 (25.0) |  |  |  |  |  |  | 1 (25.0) |  |
| **TOTAL** | **81** | 3 (3.7) | 6 (7.4) | 28 (34.6) | 23 (28.4) | 6 (7.4) | 10 (12.3) | 6 (7.4) | 2 (2.5) | 1 (1.2) | 1 (1.2) | 1 (1.2) | 3 (3.7) |
